# Assessment of the Antimicrobial Activity and the Entomocidal Potential of *Bacillus thuringiensis* Isolates from Algeria

**DOI:** 10.3390/toxins9040139

**Published:** 2017-04-13

**Authors:** Zahia Djenane, Farida Nateche, Meriam Amziane, Joaquín Gomis-Cebolla, Fairouz El-Aichar, Hassiba Khorf, Juan Ferré

**Affiliations:** 1 Microbiology Group, Laboratory of Cellular and Molecular Biology, Faculty of Biological Sciences, University of Science and Technology Houari Boumediene (USTHB), BP 32, EL ALIA, Bab Ezzouar, 16111 Algiers, Algeria; zad@uv.es (Z.D.); fnateche@yahoo.fr (F.N.); mer.amziane@gmail.com (M.A.); fifiel07@yahoo.fr (F.E.-A.); hassibakhorf@gmail.com (H.K.); 2 ERI BIOTECMED and Department of Genetics, Universitat de València, Dr. Moliner, 50, BURJASSOT, 46100 Valencia, Spain; joaquin.gomis@uv.es; 3 Department of Science and Technology, Faculty of Science, University Dr Yahia Frès, 26000 Médéa, Algeria

**Keywords:** *B. thuringiensis*, antibacterial, antifungal, *cry*, *vip3*, chitinase, biocontrol

## Abstract

This work represents the first initiative to analyze the distribution of *B. thuringiensis* in Algeria and to evaluate the biological potential of the isolates. A total of 157 isolates were recovered, with at least one isolate in 94.4% of the samples. The highest Bt index was found in samples from rhizospheric soil (0.48) and from the Mediterranean area (0.44). Most isolates showed antifungal activity (98.5%), in contrast to the few that had antibacterial activity (29.9%). A high genetic diversity was made evident by the finding of many different crystal shapes and various combinations of shapes within a single isolate (in 58.4% of the isolates). Also, over 50% of the isolates harbored *cry1*, *cry2*, or *cry9* genes, and 69.3% contained a *vip3* gene. A good correlation between the presence of chitinase genes and antifungal activity was observed. More than half of the isolates with a broad spectrum of antifungal activity harbored both endochitinase and exochitinase genes. Interestingly, 15 isolates contained the two chitinase genes and all of the above *cry* family genes, with some of them harboring a *vip3* gene as well. The combination of this large number of genes coding for entomopathogenic proteins suggests a putative wide range of entomotoxic activity.

## 1. Introduction

The economies of most countries worldwide are based on agriculture, which are threatened by various phytopathogens such as bacteria, fungi, or insects. Up to now, *B. thuringiensis* is the most used biological agent for the control of insect pests, mainly Lepidopteran species, the most injurious pests of cereals [1,2], and palms [3,4], which are the most important cultivated crops in North Africa.

*Bacillus thuringiensis* is a ubiquitous Gram positive bacterium found in various ecological habitats such as soil, sediment, stored products, dust, dead insects, phylloplane, and aquatic environments [5,6,7,8,9,10,11]. It has been the subject of most of the research and applications in the biological control of phytopathogenic insects, mainly due to the entomotoxic properties of some strains. The main interest of its use is to replace chemical pesticides with a new sustainable alternative, that is biodegradable and friendly to the environment and public health. Cry and Vip proteins, synthesized during the stationary and the vegetative phase, respectively, form the primary axis in *B. thuringiensis* based biological control of insect pests. In addition, other molecules synthesized by this bacterium can either act in synergy with Cry and Vip proteins or as an antimicrobial agent against several pathogenic and/or phytopathogenic bacteria and fungi. These could be chitinases [12,13], acylhomoserine lactone lactonase [14,15], some lipopeptides [16,17,18], and certain antibiotics such as zwittermycin [19,20].

The Cry proteins (or δ-endotoxins) accumulate during sporulation producing crystalline inclusions with several morphologies [21,22,23,24]. They exhibit specific activity against one or several orders of insects belonging to the orders Lepidoptera, Diptera, and Coleoptera [22,25,26], mainly due to the specificity of membrane receptors [27,28]. These receptors are absent in beneficial insects, plants, and mammals [26,29]. The identification of *B. thuringiensis* isolates carrying a wide variety of *cry* genes suggests a broad entomotoxic spectrum against different insect hosts [30,31].

Vip proteins are known to complement or synergize the insecticidal activities of Cry proteins [32]. They are produced by certain *B. thuringiensis* strains and bind to receptors that are different from those of Cry proteins [33,34], and thus, they have a spectrum of activity complementary to that of the Cry proteins. Therefore, a combination of Cry and Vip proteins could broaden the spectrum of insecticidal activity [35,36,37,38] and prevent the evolution of resistance of insects to Cry proteins [39,40,41].

A threat to the *B. thuringiensis-*based insecticides is the development of resistance by the insect populations exposed to them or to transgenic crops expressing their insecticidal proteins (Bt-crops) [39,42]. Therefore, the search for novel genes or new alleles encoding for insecticidal proteins, or other type of biomolecules that could synergize the action of the Cry and Vip proteins, is highly desirable.

Chitinases are enzymes that hydrolyze chitin (β-1,4-*N*-acetyl-aligned-glucosamine polymer), the main component of the invertebrates’ exoskeleton and fungi outer wall. They have been used for a long time to control several fungal pests [12,13,43,44,45], as synergistic agents to increase the entomotoxicity of biopesticides [46,47,48,49,50] and in the production of recombinant strains of *B. thuringiensis* [51,52] or transgenic plants [53,54]. Within the insect, chitinase potentiates the toxicity of the *B. thuringiensis* Cry proteins by perforating the peritrophic barrier of the midgut of the larvae, and thus, increasing the access of δ-endotoxins to the receptors located in the outer membrane of the epithelial cells [47]. The subsequent pores that are formed facilitate the penetration of spores in the hemolymph [46,48].

The aim of the present study was to screen *B. thuringiensis* isolates for the presence of a wide variety of biomolecules with the potential for insect, bacterial, and fungi control. This is the first initiative to perform a country-wide study of this bacterial species in Algeria, a Mediterranean country with a vast area (about 2382 million km^2^), large landscape diversity, and a high variability of climatic regions (Mediterranean, Sub-arid, and Desert).

## 2. Results

### 2.1. Isolation and Distribution of B. thuringiensis Isolates

**Figure 1:**
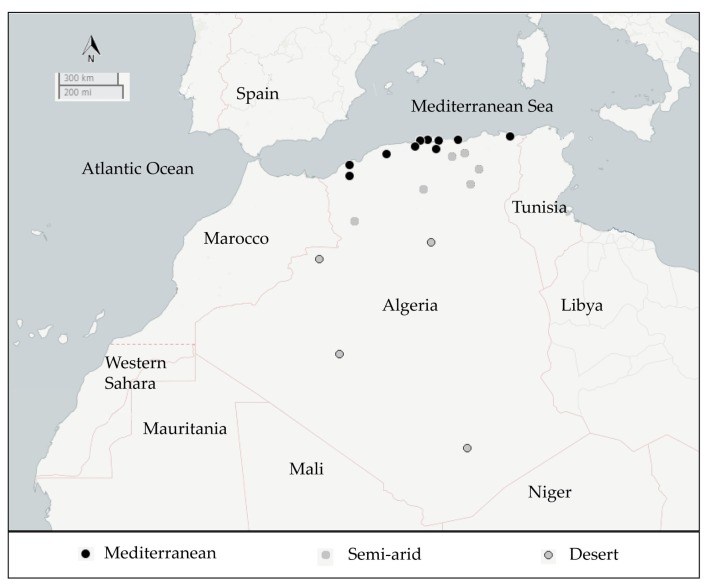
Map of Algeria showing the geographic distribution (localities) where the samples were collected (circles). The different type of circles used for the localities reflect the climatic nature of the region from which the samples were collected.

A total of 157 crystalliferous colonies (*B. thuringingiensis*) were isolated from 54 samples collected from five ecological niches (rhizospheric and non rhizospheric soil, sediment, dead insects, and grain storage) distributed over three geographical areas of Algeria viz., Mediterranean, Semi-arid, and Desert (Table 1 and Figure 1).

As shown in Table 1, *B. thuringiensis* was found in 51 (94.4%) out of the 54 collected samples. It was present with a high recovery (more than 50%) in all the ecological sources. With respect to the geographical origin, 100% of the samples collected from the Mediterranean and Semi-arid area harbored *B. thuringiensis* isolates, whereas their frequency in the Desert was 78.6%. The global Bt index was 0.41 and it varied considerably depending on the sample source. Within the different ecological niches, it ranged from 0.27 (in the non-rhizospheric soil) to 0.48 (in the rhizospheric soil). Regarding the geographical distribution, the Bt index varied from 0.32 (in samples from the Semi-arid area) to 0.44 (in samples from the Mediterranean area). The highest Bt index (0.51) was obtained with samples collected from rhizospheric soil either in the Mediterranean area or from the Desert.

From the original 157 *B. thuringingiensis* isolates, 137 were chosen for further phenotypic, biological, and molecular characterization.

### 2.2. Phenotypic Characterization of Parasporal Crystals

**Figure 2:**
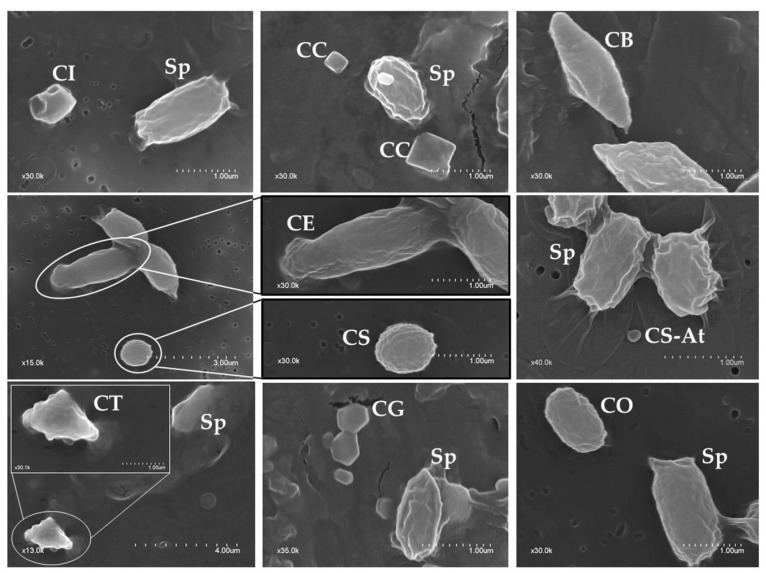
Scanning electronic microscopy (SEM) of *B. thuringiensis* isolates, showing some of the characterized parasporal inclusion shapes. Sp: spore, C: crystal (CB: bipyramidal, CC: cuboidal, CE: elongate, CG: geometrical, CI: irregular, CO: ovoid, CS: spherical, CS-At: spherical attached to the spore/sporangium, CT: triangular).

Based on the morphology of the crystalline inclusions (independent of whether they were present alone or in combination with other shapes), the isolates were classified into seven groups (Table 2). The most abundant shape was spherical (64.2% of isolates) and the least abundant one was the elongate crystal (3.6%). An example of the observed shapes is shown in Figure 2.

Regarding the number of different crystal shapes found within the same strain, 57 out of the 137 isolates (41.6%) harbored only one crystal shape, while 80 isolates (58.4%) had several shapes including 59 (43.1%) with two shapes and 21 (15.3%) having more than two shapes. We also observed that the most abundant combination was spherical-bipyramidal (10.9%) followed by spherical-geometrical (8%), spherical-triangular (4.4%), bipyramidal-geometrical (3.6%), and spherical-cuboidal (2.9%). The cuboidal and elongate crystal shapes were present only when combined with other crystal shapes.

### 2.3. Screening of the Biological Activity

#### 2.3.1. Antibacterial Activity

**Figure 3:**
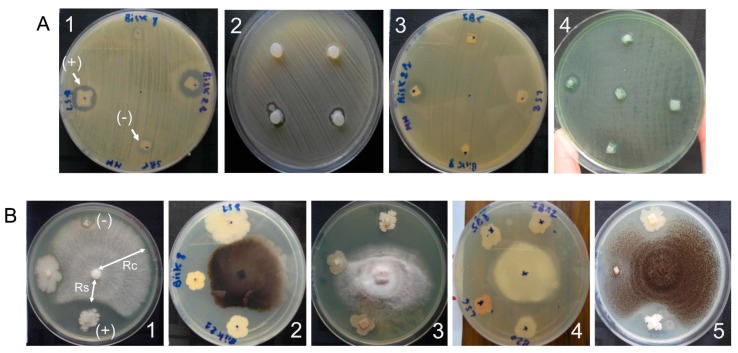
Antimicrobial activity of *B. thuringiensis* isolates. Panel (**A**): Antibacterial activity evaluated by the agar plug diffusion method. Plugs from four or five Bt isolates were tested on each Mueller Hinton Agar (MHA) plate. The pathogenic test bacteria (indicator) grew on the whole surface. A clear zone (+) around some Bt plugs indicated the presence of antibacterial activity (synthesis and diffusion of antibacterial molecules). A1: *Staphylococcus aureus* sensitive to methicillin ATCC25923, A2: *Staphylococcus aureus* resistant to methicillin ATCC34300, A3: *Escherichia coli* ATCC25922, and A4: *Pseudomonas aeruginosa* ATCC25853. Panel (**B**): Antifungal activity assay evaluated by the dual culture method. Each Potato Dextrose Agar (PDA) plate contained the fungal plug of one test fungus (center of the Petri dish) and three to four bacterial plugs (corresponding to three different Bt isolates) deposited radially 2.5 cm away. A fourth position in the plate was left empty as a negative control. The antifungal activity of the Bt isolates was revealed by the inhibition of fungal growth facing that bacterial plug as compared with the fungal growth facing the control area. The fungus grew around the plugs of bacteria that lack antifungal activity. B1: *Fusarium* sp., B2: *Monelia* sp., B3: *Coletotricum* sp., B4: *Thielaviopsis* sp., B5: *Aspergilus niger*.

*Bacillus thuringiensis* isolates were tested for their antibacterial activity against four pathogenic bacteria, two Gram positive (*Staphylococcus aureus* including a wild type variant (SM) and a resistant to methicillin variant (RM)), and two Gram negative (*Escherichia coli* and *Pseudomonas aeruginosa*) (Figure 3A). Among the 137 *B. thuringiensis* isolates, 41 (29.9%) showed activity against at least one tested pathogenic bacteria (Table 3). Considering each test bacterium independently, 30 *B. thuringiensis* isolates were active against *S. aureus* SM (21.9%), 27 isolates were active against *S. aureus* RM (19.7%), 20 against *E. coli* (14.6%), and 10 against *P. aeruginosa* (7.3%). Table 3 summarizes the combined/single antibacterial activity of those isolates.

#### 2.3.2. Antifungal Activity

The antifungal activity of *B. thuringiensis* isolates was tested against five phytopathogenic fungi (Figure 3B). Almost all isolates tested (135 out of 137) exhibited activity against at least one fungus and 81 (59%) isolates were active against at least three fungi (Table 4). Considering each test fungus independently, 106 *B. thuringiensis* isolates (77.4%) inhibited the growth of *Aspergilus niger*, 98 isolates (71.5%) were active against *Colletotricum* sp., 81 (59.1%) against *Monilia* sp., 65 (47.4%) against *Thielaviopsis* sp., and 54 (39.4%) against *Fusarium* sp. Table 4 summarizes the combined/single antifungal activity of those isolates.

### 2.4. Molecular Screening

#### 2.4.1. *cry* and *vip* Gene Families (*cry1*, *cry2*, *cry9*, and *vip3*)

**Figure 4:**
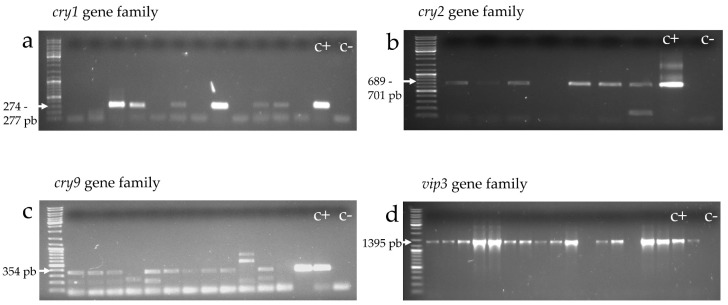
Agarose (1%) gel electrophoresis of PCR products amplified with the set of primers *Un1*(f)/*Un1*(r) (**a**), *Un2*(f)/*Un2*(r) (**b**), *Un9*(f)/*Un9*(r) (**c**), and *vip3-sc*(f)/*vip3scII*(r) (**d**), which reveal the presence of genes from the *cry1*, *cry2*, *cry9*, and *vip3* families, respectively. *Bacillus thuringiensis* isolates were considered positive for the studied gene when their genomic DNA amplified with the corresponding primers and gave a band of the expected size.

Identification of gene-families coding for lepidopteran-active toxins was carried out with universal primers used for amplifying the *cry1*, *cry2*, *cry9*, and *vip3* genes (Table 5). Isolates giving an amplicon of the expected size were considered positive to the corresponding gene-type (Figure 4). Table 6 shows that out of the 137 *B. thuringiensis* isolates, 112 (82%) were positive for at least one *cry* gene. Genes from the *cry1*, *cry2*, and *cry9* families occurred in 54%, 59.9%, and 50.4% of the isolates, respectively. The *vip3* gene was found in 95 (69.3%) of the isolates, 13 of which did not contain any other lepidopteran-active toxin gene.

#### 2.4.2. Exochitinase (*chi36*) and Endochitinase (*chit*) Genes

**Figure 5:**
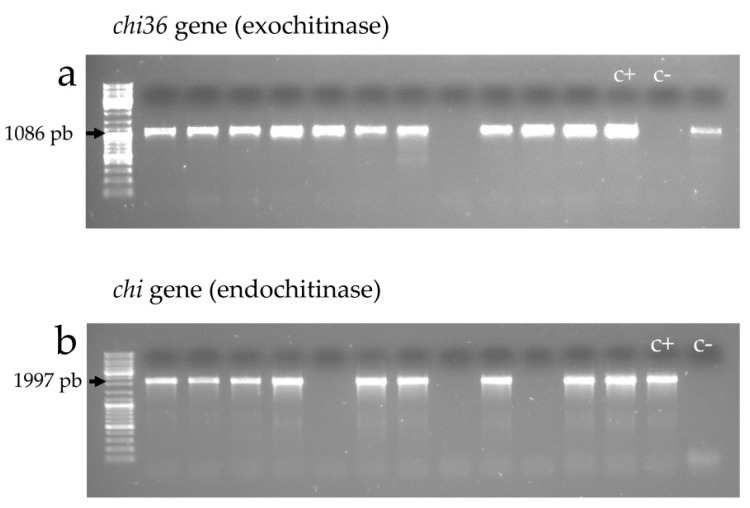
Agarose (1%) gel electrophoresis of PCR products amplified with the set of primers *chi36*(f)/*chi36*(r) (**a**) and *chit*(f)/*chit*(r) (**b**), which reveal the presence of exochitinase 36 and endochitinase genes, respectively. *Bacillus thuringiensis* isolates were considered positive for the studied gene when the genomic DNA amplified with the corresponding primers and gave a band of the expected size.

The occurrence of exochitinase and endochitinase genes was assessed by PCR amplification using gene-specific primers (Table 5 and Figure 5). Overall, 88 (64.2%) of the 137 *B. thuringiensis* isolates harbored at least one type of the chitinase gene, with 66 (48.2%) being positive for the exochitinase gene and 82 (59.9%) being positive for the endochitinase gene (Table 6). Sixty isolates (43.8%) harbored both types of genes and 28 (20.4%) exhibited only one of them.

Table 7 shows the relationship between the chitinase genes content and the spectrum of antifungal activity. Among the 81 *B. thuringiensis* isolates showing a wide spectrum against at least three fungi, 50 isolates harbored both exochitinase and endochitinase genes, and out of the 54 isolates with a narrower spectrum of antifungal activity, 27 were negative for both chitinase genes.

## 3. Discussion

The current work is the first initiative to perform a country-wide study of *B. thuringiensis* in Algeria. A collection of 157 *B. thuringiensis* isolates was built from samples collected from various niches (soil, sediment, dead insects, and grain storage) in three different climatic regions (Mediterranean, Semi-arid, and Desert). In all locations, no Bt-based biopesticide had been previously applied. Overall, 94.4% of the samples collected yielded at least one colony of *B. thuringiensis*. This high recovery reflected the large abundance of this species in Algeria. It is comparable to that found in earlier studies surveying various ecosystems, where *B. thuringiensis* recovery was over 79% [58,59,60]. Our results confirm the ubiquity of *B. thuringiensis*, since it was detected in samples from all the ecological and geographical habitats analyzed, including very arid ecosystems.

The global Bt index observed was relatively high (0.41) compared to earlier screening programs (less than 0.18) [10,58,61,62]. The Bt index differed among the different climatic regions (from 0.32 to 0.44) with the Mediterranean area being the richest source (0.44) (Table 1). It was relatively high to moderate in all niches (from 0.27 to 0.48). In agreement with earlier studies, samples from rhizospheric soil [58,60] and grain storage [61] were better sources for *B. thuringiensis* isolation (Bt index was 0.48 and 0.39, respectively). We found the non-rhizospheric soil to be the one with the lowest Bt index (0.27), also in agreement with previous studies [63,64,65,66]. This difference may be related to different factors, mainly the vegetation abundance, which constitutes a nutrient supply and an extra source of *B. thuringiensis* isolates, and also the physicochemical features of the biotope, as well as the presence of other symbiotic bacteria. In this context, several studies described the widespread presence of *B. thuringiensis* in the phylloplane [67,68,69,70]. Therefore, when performing screening of *B. thuringiensis* from soil samples, it would be important to distinguish between rhizospheric and non-rhizospheric soil samples.

The frequency values of the crystal shapes given in Table 2 refers to how often a given shape is found in the 137 *B. thuringiensis* isolates, independent of whether it was combined with other shapes or not. Despite the fact that bipyramidal crystals are generally reported to be the most abundant ones [9,10,71,72], in our collection the crystals with a spherical shape were the most abundant (64.2% of the isolates) (Table 2). The latter were found at a similar high frequency (about 40%) in studies carried out in Colombia [69] and Spain [59], but at very low frequency in other studies from Iran (5%) [10] and India (3.6%) [72]. Bipyramidal and irregular/geometrical crystal shapes were also frequent within the Algerian collection (33.6% and 40.1%, respectively). This percentage is comparable to that found in a study from India (28% and 21.5%, respectively) [72]. Triangular and cuboidal crystal shapes were present in 13% and 11.7% of our isolates, respectively. The differences in the distribution of the crystal shapes could be a consequence of the adaptation of this bacterium to the biotope.

A high percentage of our *B. thuringiensis* isolates (58.4%) produced more than one crystal shape (Table 2). This percentage is relatively high when compared to those found by Seifinejad et al. (40%) [10] and Mahadeva Swamy et al. (36%) [72]. Among the diverse combinations observed, spherical crystals were found combined with bipyramidal crystals (10.9%), geometrical crystals (8%), triangular crystals (4.4%), and cuboidal crystals (2.9%). These results demonstrated the high diversity and variability of the native *B. thuringiensis* isolates from Algeria and reflected their genetic diversity.

Some crystal shapes have been related to the expression of specific Cry proteins [24,55,68,71]. For example, the expression of *cry4*, *cry10*, or *cry11* genes give rise to spherical shape crystals, and their respective proteins are known to be active against Diptera [73,74,75,76]. Crystals with a bipyramidal shape result from the accumulation of Cry1 or Cry9 proteins, which are active mainly against Lepidoptera [24,77,78]. Cry2 proteins, some of which are active against both Lepidoptera and Diptera, form cuboidal crystals [24,77,78,79]. Therefore, the combination of several crystal shapes within an individual *B. thuringiensis* isolate, which is an indication of the presence of Cry proteins from different families, holds the potential for a spectrum of activity against a broad range of insect pests [30,31].

Overall, 29.9% of the *B. thuringiensis* isolates in our collection were active against at least one pathogenic bacterium. Three isolates inhibited all four pathogenic bacteria, including the resistant variant of *S. aureus*. This reflected a wide range of antibacterial molecules synthetized by these *B. thuringiensis* isolates, which could be further used in the control of some pathogenic and/or phytopathogenic diseases. It would be interesting to survey those isolates against some phytopathogenic bacteria causing serious losses in fruits and vegetables in Algeria, such as *Erwinia amylovora* and *Erwinia carotovora* [80,81]. In 2012, Djenane [82] investigated 97 isolates of *Bacillus* spp. and showed that the most potent *Bacillus* species in terms of antibacterial activity do not belong to the *B. thuringiensis* species, but mainly to *B. amyloliquefasiens* and *B. subtilis*. The same finding was reported by Mora et al. [83], who found that the *B. thuringiensis* species belonged to the group of plant-associated bacteria with the lowest antimicrobial activity.

It is important to note that in the reported antibacterial activity of *B. thuringiensis* isolates from our study, the activity was observed after 24 h using a fresh culture on the surface of a rich medium (MHA plates). These conditions are appropriate for bacterial growth but not for *B. thuringiensis* sporulation. Thus, some molecules synthetized during the stationary phase, and exhibiting an antibacterial activity, such as Cry11A and Cry4B [84], the 28 kDa and 37 kDa fragments from Cry1A, and the 49 kDa fragment from Cry3Aa [85], could not have contributed to the reported activity.

*Bacillus thuringiensis* isolates collected in Algeria form a good source of antifungal-specific candidates (98%) compared to the antibacterial ones (29.9%). It might be a consequence of the adaptation of this bacterium to the appropriate biotope (soil, phylloplane, grain storage, dust), where fungus proliferation is common. More than 60% of the isolates showed activity against *Monolia* sp., *Colletotricum* sp., and *A. flavus*, 47% against *Thielaviopsis* sp., and 39% against *Fusarium* sp. Moreover, 59% of the isolates exhibited broad spectrum activity against at least three phytopathogenic fungi and, among them, 24 isolates (17.5%) were active against all the five fungi tested. These high antifungal potentials could be related to a panoply of antimicrobial molecules such as zwittermycin [86], lipopeptides [17,83,87], and chitinase [12,13,43,44]. Earlier surveys showed the contribution of lipopeptides to the antifungal activitiy in some *Bacillus* species [16,83,87,88]. The latter was confirmed in *B. thuringiensis* strains from Algeria by Abderrahmani et al. [17,89]. The 24 isolates with the highest spectrum of activity could be good candidates to control fungal pests of serious economic impact in agriculture, both in North Africa and the rest of the world [90,91]. Specifically, in Algeria, the most injurious fungus species affecting palms are *Fusarium oxysporum,* the causal agent of ‘bayoud’, or Fusarium wilt [92,93], and *Thielaviopsis paradoxa*, the agent of the black scorch disease [94,95]. Different species of the genus *Fusarium* also affect cereals [2,96], forest trees (Aleppo Pine) [97,98], vegetables [99], and legumes [100]. Similar to the antibacterial activity, earlier studies showed that *B. thuringiensis* isolates were less potent, in terms of antifungal activity, compared to other *Bacillus* species such as *B. amyloliquefaciens* and *B. subtilis* [82,83].

Other than lipopeptides, chitinase enzymes exhibit a strong antifungal activity [12,13,43,44,45]. In the current work, a good correlation between the presence of both chitinase genes in *B. thuringiensis* isolates and their broad antifungal activity was observed. Essentially, more than half of the isolates (ratio 0.6) showing a broad spectrum of antifungal activity (against at least three fungi) had both chitinase genes (Table 7). These isolates would form the best candidates for fungal pest control. A synergistic activity between chitinase enzymes and other biomolecules could enhance and broaden the antifungal activity. However, it is interesting to note that 20 isolates had a broad spectrum of antifungal activity but did not exhibit any of the tested chitinase genes. Thus, possibly other chitinases and/or other antifungal molecules could be involved in that high antifungal activity.

Lepidoptera-specific insecticidal protein genes were present in a high frequency within the Algerian collection of *B. thuringiensis*: 82% of the 137 isolates harbored at least one *cry* gene, which is similar to what was found in earlier surveys investigating *cry1*, *cry2*, and *cry9* genes [10,66,101]. Every *cry* gene family was found in more than half of the isolates (54% *cry1*, 60% *cry2*, and 50% *cry9*). Among the isolates containing a *cry1* gene, 76% carried a *cry2* gene and 58% carried a *cry9* gene. Among those containing a *cry2* gene, 68% and 60% carried a *cry1* and a *cry9* gene, respectively; and among those containing a *cry9* gene, 62% and 71% carried a *cry1* and a *cry2* gene, respectively. Previous studies [9,55,62] suggested that the *cry1* and *cry2* genes are genetically associated since they occur together in a high frequency. Several complete genome sequencing programs described that many *cry* genes (most of them belonging to the *cry1* and *cry2* families) are located on the same plasmid [102,103,104,105,106]. This could also explain the pair-wise co-occurrence of the *cry1*, *cry2*, and *cry9* genes within the Algerian *B. thuringiensis* collection.

The *vip3* gene family was also present in a high percentage of the isolates (69.3%). This high frequency of *vip3* genes was previously found by Seifinejad et al. [10] (82% out of the 70 *B. thuringiensis* isolates from Iran), Yu et al. [107] (67.4% of the 2134 *B. thuringiensis* isolates from China), and Hernández-Rodríguez et al. [57] (48.9% of the 507 *B. thuringiensis* isolates from Spain).

In our study, the genetic diversity observed among isolates based on the morphological variability of crystal shapes (58.4% of the isolates harbored more than one crystal shape) correlated with the diversity in *cry* genes. Despite the fact that we studied only three *cry* gene families coding for crystals with a cuboidal shape (*cry2*) and bipyramidal/geometrical shape (*cry1* and *cry9*), 58% of *B. thuringiensis* isolates from Algeria contained more than one *cry* gene family, of which 35 (25%) contained all three studied *cry* genes.

Relating the results of the *cry* gene content with the chitinase gene content may help to select isolates with a wider spectrum of activity, since the chitinase activity was described to help synergize the effect of Cry toxins [46,47,50]. Table 6 shows that many of the isolates have a high potential for insecticidal activity because they contain a wide set of entomotoxic protein genes. Interestingly, 15 isolates contained all the three studied *cry* gene families as well as exochitinase and endochitinase genes and, among these, 11 also carried a *vip3* gene (data not shown). These isolates could be preselected as putative candidates with a high and broad spectrum of insecticidal activity due to a possible synergistic action of several insecticidal molecules. Further entomotoxic assays against a wide range of lepidopteran species would help to select the best candidate for biological control.

## 4. Conclusions

In summary, the current work showed that Algerian samples are a good source of *B. thuringiensis* isolates with potential applications in agricultural pest control. A high abundance of this species was noted within the different ecological and geographical sources. Also, a high number of isolates showed a strong activity against phytopathogenic fungi, which could be related to the role of this bacterium in its natural habitat. In addition, molecular screening evidenced the high genetic diversity of *B. thuringiensis* isolates in terms of *cry*, *vip3*, and chitinase gene content. This study lays the basis to select those *B. thuringiensis* isolates, with a wide set of entomotoxic genes, to be subjected to a screening program to evaluate their insecticidal activity in bioassays with lepidopteran pests.

## 5. Materials and Methods

### 5.1. Sample Collection

A total of 54 samples were collected from different habitats (soil, sediment, stored grains and dead insects) from 20 different locations within the Algerian territory (Table 1 and Figure 1). The source of these samples had no history of treatment with any bio-pesticide. Soil samples were collected with a sterile scraper at a depth of 10–15 cm after removing the top layer of soil. Dust or grains were collected by scooping directly from the floor or with machinery from storage. All samples including dead insects were directly transferred into sterile plastic bags and stored at 4 °C until processed.

### 5.2. Reference Strains

The pathogenic bacteria used for the antibacterial test belonged to the American Type Collection Culture. The species and strains used were *Pseudomonas aeruginosa* ATCC25853 (*P. aeruginosa*), *Escherichia coli* ATCC25922 (*E. coli*), *Staphylococcus aureus* sensitive to methicillin ATCC25923 (*S. aureus* SM), and *Staphylococcus aureus* resistant to methicillin ATCC34300 (*S. aureus* RM).

The phytopathogenic fungi, used for the antifungal test, were kindly provided by the Algerian National Institute for Plant Protection (*Fusarium* sp., *Colletotrichum* sp., *Monilia* sp., *Thielaviopsis* sp., and *Aspergilus niger*).

### 5.3. Bacillus Thuringiensis Culturing and Isolation

Isolation of *B. thuringiensis* was carried out according to the method of Travers et al. [108] with slight modifications. One gram from each sample was suspended in 9 mL sterile physiological solution (0.9% NaCl). This stock solution was heated at 70 °C for 10 min and then used to prepare 10^−1^, 10^−2^, and 10^−3^ dilutions. An aliquot (100 µL) of each solution was spread onto three Nutrient Agar (NA) plates. The plates were incubated at 30 °C for at least 3 days. The preselected *Bacillus* like-colonies (whitish, not bright, flat, dry, rough surface, and irregular border) were examined by phase-contrast microscopy. Only colonies containing bacillary cells producing spores and crystals (phase-bright inclusions) were selected as *B. thuringiensis*. Within the same sample, when colonies showed a similar macroscopic and/or microscopic aspect, only one colony was selected. Thereby, we reduced the number of sibling strains and avoided duplicates. The selected *B. thuringiensis* colonies were plated again for single-colony purification and stored at −20 °C in 20% and 50% glycerol medium. The Bt index was defined as the number of crystalliferous colonies as a fraction of *Bacillus*-like colonies in a sample; it serves as an estimation of the success in *B. thuringiensis* isolation and depends on the isolation procedure as well as the sampled material [59]. Since SDS-PAGE or Western blot was not performed, it cannot be ruled out that some of the observed parasporal inclusions are non-proteinaceous.

### 5.4. Screening for Antibacterial Activity with the Agar Plug Diffusion Method

The presence of antibacterial activity was tested using a technique similar to that used in the disk-diffusion method [109,110], which is based on the NCCLS diffusion method [111]. The target bacteria (*S. aureus*, *P. aeruginosa*, and *E. coli*) were inoculated on the surface of NA plates and incubated at 37 °C for 24–48 h. Then, three to five isolated colonies were suspended in saline (physiological water 0.9%). The turbidity of the test suspension was adjusted to 0.5 McFarland turbidity standard (corresponding to 1.5 × 10^8^ CFU mL^−1^), and used as an inoculum within the following 15 min. On the surface of Mueller Hinton Agar (MHA) plates (4 mm of depth), the suspension was spread by swabbing. The *B. thuringiensis* agar-plugs were cut aseptically from pre-inoculated NA plates (4 mm depth) after 24 h of incubation at 30 °C, using a sterile cork borer. Four agar-plugs, containing a single colony each and corresponding to four different *B. thuringiensis* isolates, were transferred onto the surface of MHA plates. The antibacterial activity was observed by the appearance of a growth inhibition zone around the *B. thuringiensis* agar-plug (Figure 3A) and, for comparison purposes, it was expressed as the diameter of the inhibition zone measured after 24 h of incubation at 37 °C.

### 5.5. Screening for the Antifungal Activity

The antifungal activity was tested using the dual culture method [110,112] with slight modifications. Each fungal strain was spot-inoculated on Potato Dextrose Agar (PDA) plates and incubated for 7 days at 28 °C. A series of six mm diameter plugs were cut out from these fungal cultures (test fungi) using a sterile cork borer. Similarly, 6 mm *B. thuringiensis* plugs containing a single colony (tested bacterium) were obtained from pre-inoculated NA plates as described in the antibacterial activity method. The dual culture method consists on culturing both fungal and bacterial plugs together under the appropriate conditions of the fungal strains.

On the surface of PDA plates, fungal and bacterial plugs were aseptically transferred using a sterile toothpick. The fungal plug of one test fungus was placed at the center of the plate and three bacterial test plugs, corresponding to three different *B. thuringiensis* isolates, were deposited radially 2.5 cm away, leaving a fourth position in the plate empty as a negative control. After incubation at 28 °C for 3 to 7 days, the radius of fungal growth facing the bacterial plug or control position was measured. The antifungal effect of the *B. thuringiensis* isolates (Figure 3B) was estimated by the “inhibition radius” (IR), which is inversely proportional to the antifungal potency. The IR is defined as Rs/Rc, where, Rs and Rc correspond to the fungal growth facing the tested bacterium (*B. thuringiensis* isolates) and the control position, respectively (Figure 3B1).

### 5.6. DNA Extraction and PCR Analysis

Total DNA from *B. thuringiensis* isolates was extracted following the method described by Ferrandis et al. [113]. The polymerase chain reaction (PCR) was used for the screening of endo-chitinase, exo-chitinase, and lepidopteran-active protein coding genes *cry1*, *cry2*, *cry9*, and *vip3*. Each amplification process was performed in a 25 µL reaction mixture containing 1.0 U of *Taq* DNA polymerase (BIOTOOLS B&M Labs, S.A., Madrid, Spain), 1× *Taq* polymerase buffer, 0.4 µM of each primer, 2.5 mM MgCl_2_, 0.2 mM of dNTPs, and 1.0 µL of DNA template (about 100 ng/µL). All PCR reactions were performed in an Eppendorf Mastercycler thermal cycler (Eppendorf AG, Barkhausenweg, Germany). The amplification protocol consisted of an initial denaturation step of 4 min at 94 °C, 35 cycles of denaturation (94 °C for 40 s), annealing (50 °C for 1 min for *cry2*, *vip3*, and exochitinase, 50 °C for 45 s for *cry9* and endochitinase, and 48 °C for 50 s for *cry1*), and extension (72 °C for 1–2 min), and a final extension step at 72 °C for 7 min. PCR products were analyzed in a 1% agarose gel containing 0.5 µg/mL ethidium bromide. Primers used for the molecular screening were selected from previous studies, except the *vip3* reverse primer, which was designed from a conserved region (from 1442 to 1472) based on the alignment of previously published sequences of *vip3* genes [114]. Primers’ sequence, melting temperature, and expected amplicon size are shown in Table 5.

## Figures and Tables

**Table 1:** Description of the origin of *B. thuringiensis* isolates and the samples from where they were isolated.

Source of Samples	Samples		Mediterranean Area			Semi-Arid Area			Desert			Global Bt Index
			No. of Isolates		Bt Index ^d^	No. of Isolates		Bt Index ^d^	No. of Isolates		Bt Index ^d^	
	Total Analyzed	Bt Positive ^a^	*Bacillus*-Like ^b^	Bt ^c^		*Bacillus*-Like ^b^	Bt ^c^		*Bacillus*-Like ^b^	Bt ^c^		
Telluric (soil)												
Rhizospheric	18	18	68	35	0.51	39	14	0.36	77	39	0.51	0.48
Non rhizospheric	10	8	11	4	0.36	12	2	0.17	43	12	0.28	0.27
Non telluric												
Sediment	3	2	13	5	0.38	0	0	/	1	0	0	0.36
Dead insects	4	4	28	10	0.36	0	0	/	0	0	/	0.36
Grain storage	19	19	62	26	0.42	31	10	0.32	0	0	/	0.39
Total	54	51	182	80	0.44 ^e^	82	26	0.32 ^e^	121	51	0.42 ^e^	0.41 ^f^

^a^ Sample with at least one *B. thuringiensis* colony; ^b^ Colonies examined by microscopy; ^c^ Crystaliferous colonies identified as *B. thuringiensis*; ^d^ *B. thuringiensis* as a fraction of *Bacillus*-like isolates; ^e^ Global Bt index in each geographic area; ^f^ Global Bt index of *B. thuringiensis* collection.

**Table 2:** Description of the crystal shape variability in *B. thuringiensis* isolates.

Crystal Shape	No. of Isolates Containing Crystals with a Given Shape		
	Alone	Combined with Other Crystals	Total (%)
Spherical	30	58	88 (64.2%)
Bipyramidal	4	42	46 (33.6%)
Irregular/Geometrical	19	36	55 (40.1%)
Triangular	2	16	18 (13.1%)
Cuboidal	0	16	16 (11.7%)
Ovoid	2	8	10 (7.3%)
Elongate	0	5	5 (3.6%)

**Table 3:** Profile of the antibacterial activity of *B. thuringienis* isolates.

Spectrum of Activity	Gram Positive ^a^		Gram Negative ^b^		
	SaSM	SaRM	Ec	Pa	*n* ^c^
Against both Gram positive and Gram negative pathogenic bacteria (*n* = 20)	+	+	+	+	3
	+	+	+	−	9
	+	+	−	+	4
	+	−	+	−	2
	−	+	+	−	2
Against Gram positive pathogenic bacteria (*n* = 14)	+	+	−	−	7
	+	−	−	−	5
	−	+	−	−	2
Against Gram negative pathogenic bacteria (*n* = 7)	−	−	+	−	4
	−	−	−	+	3
Total Bt isolates positive for each bacterium type	30	27	20	10	

^a^ SaSM: *S. aureus* sensitive to methicillin ATCC25923; SaRM: *S. aureus* resistant to methicillin ATCC34300; ^b^ Ec: *E. coli* ATCC25922; Pa: *P. aeruginosa* ATCC25853; ^c^ Number of *B. thuringiensis* isolates with activity against pathogenic bacteria within the reported profile.

**Table 4:** Profile of the antifungal activity of *B. thuringiensis* isolates.

Spectrum of Activity	*Fusarium* sp.	*Monilia* sp.	*Colletotricum* sp.	*Thielaviopsis* sp.	*Aspergilus flavus*	*n* ^a^
Against five fungi (*n* = 24)	+	+	+	+	+	24
Against four fungi (*n* = 32)	+	+	+	+	−	4
	+	+	+	−	+	5
	+	+	−	+	+	9
	+	−	+	+	+	4
	−	+	+	+	+	10
Against three fungi (*n* = 25)	+	+	−	−	+	2
	−	−	+	+	+	2
	+	+	−	+	−	3
	−	+	+	+	−	2
	−	+	−	+	+	2
	+	−	−	+	+	1
	−	+	+	−	+	12
	+	−	+	−	+	1
Against two fungi (*n* = 27)	+	+	−	−	−	1
	−	+	−	−	+	5
	−	+	+	−	−	1
	−	−	+	+	−	3
	−	−	−	+	+	1
	−	−	+	−	+	16
Against one fungus (*n* = 27)	−	+	−	−	−	1
	−	−	+	−	−	14
	−	−	−	−	+	12
Total Bt isolates positive for each fungus type	54	81	98	65	106	

^a^ Number of *B. thuringiensis* isolates with antifungal activity within the reported profile.

**Table 5:** Primers used in the PCR analysis of *cry1*, *cry2*, *cry9*, *vip3*, *chi36*, and *chit* genes.

Target Gene Family	Product Size (pb)	Primers Set	Sequence (5′ → 3′)	T_m_^a^ (°C)	Reference
*cry1*	274–277	*Un1*(f)	CATGATTCATGCGGCAGATAAAC	67.2	[55]
		*Un1*(r)	TTGTGACACTTCTGCTTCCCATT	66.7	
*cry2*	689–701	*Un2*(f)	GTTATTCTTAATGCAGATGAATGGG	63.3	[55]
		*Un2*(r)	CGGATAAAATAATCTGGGAAATAGT	61.1	
*cry9*	354	*Un9*(f)	CGGTGTTACTATTAGCGAGGGCGG	71.5	[55]
		*Un9*(r)	GTTTGAGCCGCTTCACAGCAATCC	73.3	
*endochitinase*	1997	*Chit*(f)	ATTCACACTGCTATTACTATC	50	[56]
		*Chit*(r)	TGACGGCATTTAAAAGTTCGGC	68.7	
*exochitinase 36*	1083	*Chi36*(f)	GATGTTAAACAGGTTCAA	50.2	[12]
		*Chi36*(r)	TTATTTTTGCAAGGAAAG	52.9	
*vip3*	1395	*vip3-sc*(f)	TGCCACTGGTATCAARGA	54.2	[57]
		*vip3-scII*(r)	CCATTAATYGGAKTCAAAAATGTTTCACTGAT	71.1	The current work

^a^ Melting temperature.

**Table 6:** Description of the gene content of *B. thuringiensis* isolates for *cry1*, *cry2*, *cry9*, *vip3*, exochitinase (*chi36*), and endochitinase (*chit*) genes.

Presence/Absence of *cry* Gene Families	No. of Bt for Each *cry* Gene Profile	No. of Bt with a *vip3* Gene	No. of Bt with Both *chi36* and *chit*	No. of Bt with *chi36* Only	No. of Bt with *chit* Only	No. of Bt without *chi36* and *chit*
I. One *cry* gene family						
*cry1*	10	6	1	0	3	6
*cry2*	12	10	1	1	2	8
*cry9*	12	6	11	0	0	1
II. Two *cry* gene families						
*cry1* + *cry2*	21	18	6	0	5	10
*cry1* ± *cry9*	8	5	4	0	3	1
*cry2* + *cry9*	14	8	11	1	2	0
III. Three *cry* gene families						
*cry1* + *cry2* + *cry9*	35	29	15	4	4	12
IV. No *cry* gene	25	13	11	0	3	11
Total Bt isolates (%)	137	95 (69.3%)	60 (43.8%)	6 (4.4%)	22 (16.1%)	49 (35.8%)

**Table 7:** Relationship between the chitinase genes profile and the spectrum of antifungal activity.

Spectrum of the Antifungal Activity		Profile of Chitinase Genes							
		Both *chi36 and chit* ^a^		Only *chi36*		Only *chit*		None	
	*N*	*n*	*x*	*n*	*x*	*n*	*x*	*n*	*x*
Activity against at least three fungi	81	50	0.62	5	0.06	4	0.05	22	0.27
Activity against one or two fungi	54	10	0.19	1	0.02	16	0.30	27	0.5

*N*, *n*: number of *B. thuringiensis* isolates; *x*: ratio *n*/*N*. ^a^ *chit*: endochitinase.

## References

[1] Kfir R., Overholt W.A., Khan Z.R., Polaszek A. (2002). Biology and management of economically important lepidopteran cereal stem borers in Africa. Annu. Rev. Entomol..

[2] Midega C.A.O., Bruce T.J.A., Pickett J.A., Khan Z.R. (2015). Ecological management of cereal stemborers in African smallholder agriculture through behavioural manipulation. Ecol. Entomol..

[3] Gitau C.W., Gurr G.M., Dewhurst C.F., Fletcher M.J., Mitchell A. (2009). Insect pests and insect-vectored diseases of palms. Aust. J. Entomol..

[4] El-Shafie H. (2012). Review: List of arthropod pests and their natural enemies identified worldwide on date palm, *Phoenix dactylifera* L.. Agric. Biol. J. N. Am..

[5] Meadows M.P., Ellis D.J., Butt J., Jarrett P., Burges H.D. (1992). Distribution, frequency, and diversity of *Bacillus thuringiensis* in an animal feed mill. Appl. Environ. Microbiol..

[6] Ohba M., Aratake Y. (1994). Comparative study of the frequency and flagellar serotype flora of *Bacillus thuringiensis* in soils and silkworm-breeding environments. J. Appl. Bacteriol..

[7] Iriarte J., Porcar M., Lecadet M.M., Caballero P. (2000). Isolation and characterization of *Bacillus thuringiensis* strains from aquatic environments in Spain. Curr. Microbiol..

[8] Lee D.H., Machii J., Ohba M. (2002). High frequency of *Bacillus thuringiensis* in feces of herbivorous animals maintained in a zoological garden in Japan. Appl. Entomol. Zool..

[9] Hernández-Rodríguez C.S., Ferré J. (2008). Ecological distribution and characterization of four collections of *Bacillus thuringiensis* strains. J. Basic Microbiol..

[10] Seifinejad A., Jouzani G.R.S., Hosseinzadeh A., Abdmishani C. (2008). Characterization of Lepidoptera-active *cry* and *vip* genes in Iranian *Bacillus thuringiensis* strain collection. Biol. Control..

[11] Baig D.N., Mehnaz S. (2010). Determination and distribution of *cry*-type genes in halophilic *Bacillus thuringiensis* isolates of Arabian Sea sedimentary rocks. Microbiol. Res..

[12] Arora N., Ahmad T., Rajagopal R., Bhatnagar R.K. (2003). A constitutively expressed 36 kDa exochitinase from *Bacillus thuringiensis* HD-1. Biochem. Biophys. Res. Commun..

[13] Liu D., Cai J., Xie C.C., Liu C., Chen Y.H. (2010). Purification and partial characterization of a 36-kDa chitinase from *Bacillus thuringiensis* subsp. *colmeri*, and its biocontrol potential. Enzym. Microb. Technol..

[14] Lee S.J., Park S.Y., Lee J.J., Yum D.Y., Koo B.T., Lee J.K. (2002). Genes encoding the N-acyl homoserine lactone-degrading enzyme are widespread in many subspecies of *Bacillus thuringiensis*. Appl. Environ. Microbiol..

[15] Zhou Y., Choi Y.L., Sun M., Yu Z. (2008). Novel roles of *Bacillus thuringiensis* to control plant diseases. Appl. Microbiol. Biotechnol..

[16] Ongena M., Jacques P. (2008). *Bacillus* lipopeptides: Versatile weapons for plant disease biocontrol. Trends Microbiol..

[17] Abderrahmani A., Tapi A., Nateche F., Chollet M., Leclère V., Wathelet B., Hacene H., Jacques P. (2011). Bioinformatics and molecular approaches to detect NRPS genes involved in the biosynthesis of kurstakin from *Bacillus thuringiensis*. Appl. Microbiol. Biotechnol..

[18] Ben Khedher S., Boukedi H., Dammak M., Kilani-Feki O., Sellami-Boudawara T., Abdelkefi-Mesrati L., Tounsi S. (2017). Combinatorial effect of *Bacillus amyloliquefaciens* AG1 biosurfactant and *Bacillus thuringiensis* Vip3Aa16 toxin on *Spodoptera littoralis* larvae. J. Invertebr. Pathol..

[19] Broderick N.A., Goodman R.M., Raffa K.F., Handelsman J. (2000). Synergy between Zwittermicin A and *Bacillus thuringiensis* subsp. *kurstaki* against gypsy moth (Lepidoptera: Lymantriidae). Environ. Entomol..

[20] Zhao C., Luo Y., Song C., Liu Z., Chen S., Yu Z., Sun M. (2007). Identification of three Zwittermicin A biosynthesis-related genes from *Bacillus thuringiensis* subsp. *kurstaki* strain YBT-1520. Arch. Microbiol..

[21] Crickmore N., Zeigler D.R., Feitelson J., Schnepf E., Van Rie J., Lereclus D., Baum J., Dean D.H. (1998). Revision of the nomenclature for the *Bacillus thuringiensis* pesticidal crystal proteins. Microbiol. Mol. Biol. Rev..

[22] Schnepf E., Crickmore N., Van Rie J., Lereclus D., Baum J., Feitelson J., Zeigler D.R., Dean D.H. (1998). *Bacillus thuringiensis* and its pesticidal crystal proteins. Microbiol. Mol. Biol. Rev..

[23] Deng C., Peng Q., Song F., Lereclus D. (2014). Regulation of *cry* gene expression in *Bacillus thuringiensis*. Toxins (Basel).

[24] Höfte H., Whiteley H.R. (1989). Insecticidal crystal proteins of *Bacillus thuringiensis*. Microbiol. Rev..

[25] Van Frankenhuyzen K. (2009). Insecticidal activity of *Bacillus thuringiensis* crystal proteins. J. Invertebr. Pathol..

[26] Bravo A., Likitvivatanavong S., Gill S.S., Soberón M. (2011). *Bacillus thuringiensis*: A story of a successful bioinsecticide. Insect Biochem. Mol. Biol..

[27] Xu C., Wang B.C., Yu Z., Sun M. (2014). Structural insights into *Bacillus thuringiensis Cry*, *Cyt and* parasporin toxins. Toxins.

[28] Jurat-Fuentes J.L., Crickmore N. (2016). Specificity determinants for Cry insecticidal proteins: Insights from their mode of action. J. Invertebr. Pathol..

[29] Mendelsohn M., Kough J., Vaituzis Z., Matthews K. (2003). Are Bt crops safe?. Nat. Biotechnol..

[30] Chen M.L., Chen P.H., Pang J.C., Lin C.W., Hwang C.F., Tsen H.Y. (2014). The correlation of the presence and expression levels of *cry* genes with the insecticidal activities against *Plutella xylostella* for *Bacillus thuringiensis* Strains. Toxins (Basel).

[31] Monnerat R., Pereira E., Teles B., Martins E., Praça L., Queiroz P., Soberón M., Bravo A., Ramos F., Soares C.M. (2014). Synergistic activity of *Bacillus thuringiensis* toxins against *Simulium* spp. larvae. J. Invertebr. Pathol..

[32] Chakroun M., Banyuls N., Bel Y., Escriche B., Ferré J. (2016). Bacterial vegetative insecticidal proteins (Vip) from entomopathogenic bacteria. Microbiol. Mol. Biol. Rev..

[33] Sena J.A.D., Hernández-Rodríguez C.S., Ferré J. (2009). Interaction of *Bacillus thuringiensis* Cry1 and Vip3A proteins with *Spodoptera frugiperda* midgut binding sites. Appl. Environ. Microbiol..

[34] Chakroun M., Ferré J. (2014). In vivo and in vitro binding of Vip3Aa to *Spodoptera frugiperda* midgut and characterization of binding sites by ^125^I radiolabeling. Appl. Environ. Microbiol..

[35] Hernández-Martínez P., Hernández-Rodríguez C.S., Van Rie J., Escriche B., Ferré J. (2013). Insecticidal activity of Vip3Aa, Vip3Ad, Vip3Ae, and Vip3Af from *Bacillus thuringiensis* against lepidopteran corn pests. J. Invertebr. Pathol..

[36] Palma L., de Escudero I.R., Maeztu M., Caballero P., Muñoz D. (2013). Screening of *vip* genes from a Spanish *Bacillus thuringiensis* collection and characterization of two Vip3 proteins highly toxic to five lepidopteran crop pests. Biol. Control..

[37] Lemes A.R.N., Davolos C.C., Legori P.C.B.C., Fernandes O.A., Ferré J., Lemos M.V.F., Desiderio J.A. (2014). Synergism and antagonism between *Bacillus thuringiensis* Vip3A and Cry1 proteins in *Heliothis virescens*, *Diatraea saccharalis* and *Spodoptera frugiperda*. PLoS ONE.

[38] Gomis-Cebolla J., Ruiz de Escudero I., Vera-Velasco N.M., Hernández-Martínez P., Hernández-Rodríguez C.S., Ceballos T., Palma L., Escriche B., Caballero P., Ferré J. (2017). Insecticidal spectrum and mode of action of the *Bacillus thuringiensis* Vip3Ca insecticidal protein. J. Invertebr. Pathol..

[39] Ferre J., Van Rie J. (2002). Biochemistry and genetics of insect resistance to *Bacillus thuringiensis*. Annu. Rev. Entomol..

[40] Bravo A., Soberón M. (2008). How to cope with insect resistance to Bt toxins?. Trends Biotechnol..

[41] Pardo-López L., Muñoz-Garay C., Porta H., Rodríguez-Almazán C., Soberón M., Bravo A. (2009). Strategies to improve the insecticidal activity of Cry toxins from *Bacillus thuringiensis*. Peptides.

[42] Tabashnik B.E., Van Rensburg J.B.J., Carrière Y. (2009). Field-evolved insect resistance to Bt crops: Definition, theory, and data. J. Econ. Entomol..

[43] Ghasemi S., Ahmadian G., Sadeghi M., Zeigler D.R., Rahimian H., Ghandili S., Naghibzadeh N., Dehestani A. (2011). First report of a bifunctional chitinase/lysozyme produced by *Bacillus pumilus* SG2. Enzym. Microb. Technol..

[44] Hjort K., Presti I., Elväng A., Marinelli F., Sjöling S. (2014). Bacterial chitinase with phytopathogen control capacity from suppressive soil revealed by functional metagenomics. Appl. Microbiol. Biotechnol..

[45] El Guilli M., Hamza A., Clément C., Ibriz M., Ait Barka E. (2016). Effectiveness of postharvest treatment with chitosan to control citrus green mold. Agriculture.

[46] Regev A., Keller M., Strizhov N., Sneh B., Prudovsky E., Chet I., Ginzberg I., Koncz-Kalman Z., Koncz C., Schell J., Zilberstein A. (1996). Synergistic activity of a *Bacillus thuringiensis* delta-endotoxin and a bacterial endochitinase against *Spodoptera littoralis* larvae. Appl. Environ. Microbiol..

[47] Sampson M.N., Gooday G.W. (1998). Involvement of chitinases of *Bacillus thuringiensis* during pathogenesis in insects. Microbiology.

[48] Wiwat C., Thaithanun S., Pantuwatana S., Bhumiratana A. (2000). Toxicity of chitinase-producing *Bacillus thuringiensis* ssp. *kurstaki* HD-1 (G) toward *Plutella xylostella*. J. Invertebr. Pathol..

[49] Sirichotpakorn N., Rongnoparut P., Choosang K., Panbangred W. (2001). Coexpression of chitinase and the *cry11Aa1* toxin genes in *Bacillus thuringiensis* serovar *israelensis*. J. Invertebr. Pathol..

[50] Barboza-Corona J.E., Ortiz-Rodríguez T., de la Fuente-Salcido N., Bideshi D.K., Ibarra J.E., Salcedo-Hernández R. (2009). Hyperproduction of chitinase influences crystal toxin synthesis and sporulation of *Bacillus thuringiensis*. Antonie Van Leeuwenhoek.

[51] Lertcanawanichakul M., Wiwat C., Bhumiratana A., Dean D.H. (2004). Expression of chitinase-encoding genes in *Bacillus thuringiensis* and toxicity of engineered *B. thuringiensis* subsp. *aizawai* toward *Lymantria dispar* larvae. Curr. Microbiol..

[52] Driss F., Rouis S., Azzouz H., Tounsi S., Zouari N., Jaoua S. (2011). Integration of a recombinant chitinase into *Bacillus thuringiensis* parasporal insecticidal crystal. Curr. Microbiol..

[53] Broglie R., Broglie K. (1993). Chitinase gene expression in transgenic plants: A molecular approach to understanding plant defence responses. Philos. Trans. R. Soc. B Biol. Sci..

[54] Cletus J., Balasubramanian V., Vashisht D., Sakthivel N. (2013). Transgenic expression of plant chitinases to enhance disease resistance. Biotechnol. Lett..

[55] Ben-Dov E., Zaritsky A., Dahan E., Barak Z., Sinai R., Manasherob R., Khamraev A., Troitskaya E., Dubitsky A., Berezina N. (1997). Extended screening by PCR for seven *cry*-group genes from field-collected strains of *Bacillus thuringiensis*. Appl. Environ. Microbiol..

[56] Raddadi N., Belaouis A., Tamagnini I., Hansen B.M., Hendriksen N.B., Boudabous A., Cherif A., Daffonchio D. (2009). Characterization of polyvalent and safe *Bacillus thuringiensis* strains with potential use for biocontrol. J. Basic Microbiol..

[57] Hernández-Rodríguez C.S., Boets A., Van Rie J., Ferré J. (2009). Screening and identification of *vip* genes in *Bacillus thuringiensis* strains. J. Appl. Microbiol..

[58] Bel Y., Granero F., Alberola T.M., Martínez-Sebastián M.J., Ferré J. (1997). Distribution, frequency and diversity of *Bacillus thuringiensis* in olive tree environments in Spain. Syst. Appl. Microbiol..

[59] Vidal-Quist J.C., Castañera P., González-Cabrera J. (2009). Diversity of *Bacillus thuringiensis* strains isolated from citrus orchards in Spain and evaluation of their insecticidal activity against *Ceratitis capitata*. J. Microbiol. Biotechnol..

[60] Alper M., Güneş H., Tatlipinar A., Çöl B., Civelek H.S., Özkan C., Poyraz B. (2014). Distribution, occurrence of *cry* genes, and lepidopteran toxicity of native *Bacillus thuringiensis* isolated from fig tree environments in Aydän Province. Turk. J. Agric. For..

[61] Ejiofor A.O., Johnson T. (2002). Physiological and molecular detection of crystalliferous *Bacillus thuringiensis* strains from habitats in the South Central United States. J. Ind. Microbiol. Biotechnol..

[62] Wang J., Boets A., Van Rie J., Ren G. (2003). Characterization of *cryl*, *cry2*, and *cry9* genes in *Bacillus thuringiensis* isolates from China. J. Invertebr. Pathol..

[63] DeLucca A.J., Simonson J.G., Larson A.D. (1981). *Bacillus thuringiensis* distribution in soils of the United States. Can. J. Microbiol..

[64] Ohba M., Aizawa K. (1986). Distribution of *Bacillus thuringiensis* in soils of Japan. J. Invertebr. Pathol..

[65] Ramalakshmi A., Udayasuriyan V. (2010). Diversity of *Bacillus thuringiensis* isolated from Western Ghats of Tamil Nadu State, India. Curr. Microbiol..

[66] Asokan R., Mahadeva Swamy H.M., Thimmegowda G.G., Mahmood R. (2013). Diversity analysis and characterization of Coleoptera, Hemiptera and Nematode active *cry* genes in native isolates of *Bacillus thuringiensis*. Ann. Microbiol..

[67] Smith R.A., Couche G.A. (1991). The phylloplane as a source of *Bacillus thuringiensis* variants. Appl. Environ. Microbiol..

[68] Mizuki E., Ichimatsu T., Hwang S.H., Park Y.S., Saitoh H., Higuchi K., Ohba M. (1999). Ubiquity of *Bacillus thuringiensis* on phylloplanes of arboreous and herbaceous plants in Japan. J. Appl. Microbiol..

[69] Maduell P., Callejas R., Cabrera K.R., Armengol G., Orduz S. (2002). Distribution and Characterization of *Bacillus thuringiensis* on the phylloplane of species of piper (Piperaceae) in three altitudinal levels. Microb. Ecol..

[70] Jara S., Maduell P., Orduz S. (2006). Diversity of *Bacillus thuringiensis* strains in the maize and bean phylloplane and their respective soils in Colombia. J. Appl. Microbiol..

[71] Rosas-García N.M., Mireles-Martínez M., Hernández-Mendoza J.L., Ibarra J.E. (2008). Screening of *cry* gene contents of *Bacillus thuringiensis* strains isolated from avocado orchards in Mexico, and their insecticidal activity towards *Argyrotaenia* sp. (Lepidoptera: Tortricidae) larvae. J. Appl. Microbiol..

[72] Mahadeva Swamy H.M., Asokan R., Mahmood R., Nagesha S.N. (2013). Molecular characterization and genetic diversity of insecticidal crystal protein genes in native *Bacillus thuringiensis* isolates. Curr. Microbiol..

[73] Saitoh H., Higuchi K., Mizuki E., Hwang S.H., Ohba M. (1998). Characterization of mosquito larvicidal parasporal inclusions of a *Bacillus thuringiensis* serovar *higo* strain. J. Appl. Microbiol..

[74] Aboussaid H., Vidal-Quist J.C., Oufdou K., El Messoussi S., Castañera P., González-Cabrera J. (2011). Occurrence, characterization and insecticidal activity of *Bacillus thuringiensis* strains isolated from argan fields in Morocco. Environ. Technol..

[75] Mahalakshmi A., Sujatha K., Kani P., Shenbagarathai R. (2012). Distribution of *cry* and *cyt* genes among indigenous *Bacillus thuringiensis* isolates with mosquitocidal activity. Adv. Microbiol..

[76] El-Kersh T.A., Ahmed A.M., Al-Sheikh Y.A., Tripet F., Ibrahim M.S., Metwalli A.A.M. (2016). Isolation and characterization of native *Bacillus thuringiensis* strains from Saudi Arabia with enhanced larvicidal toxicity against the mosquito vector *Anopheles gambiae* (s.l.). Parasit Vectors.

[77] Ibarra J.E., Federici B.A. (1986). Parasporal bodies of *Bacillus thuringiensis* subsp. *morrisoni* (PG-14) and *Bacillus thuringiensis* subsp. *israelensis* are similar in protein composition and toxicity. FEMS Microbiol. Lett..

[78] Samasanti W., Tojo A., Aizawa K. (1986). Insecticidal activity of bipyramidal and cuboidal inclusions of delta-endotoxin and distribution of their antigens among various strains of *Bacillus thuringiensis*. Agric. Biol. Chem..

[79] López-Meza J.E., Ibarra J.E. (1996). Characterization of a novel strain of *Bacillus thuringiensis*. Appl. Environ. Microbiol..

[80] Serfontein S., Logan C., Swanepoel A.E., Boelema B.H., Theron D.J. (1991). A potato wilt disease in South Africa caused by *Erwinia carotovora* subspecies *carotovora* and *E. chrysanthemi*. Plant Pathol..

[81] Jock S., Völksch B., Mansvelt L., Geider K. (2002). Characterization of *Bacillus* strains from apple and pear trees in South Africa antagonistic to *Erwinia amylovora*. FEMS Microbiol. Lett..

[82] Djenane Z. (2012). Criblage de Souches Autochtones de *Bacillus* en vue de la Mise en Evidence de Molécules Actives Présentant un Intérêt En Biotechnologie Industrielle Et Santé. Master’s Thesis.

[83] Mora I., Cabrefiga J., Montesinos E. (2015). Cyclic lipopeptide biosynthetic genes and products, and inhibitory activity of plant associated *Bacillus against* phytopathogenic bacteria. PLoS ONE.

[84] Yudina T.G., Konukhova A.V., Revina L.P., Kostina L.I., Zalunin I.A., Chestukhina G.G. (2003). Antibacterial activity of Cry and Cyt proteins from *Bacillus thuringiensis* ssp. *israelensis*. Can. J. Microbiol..

[85] Yudina T.G., Brioukhanov A.L., Zalunin I.A., Revina L.P., Shestakov A.I., Voyushina N.E., Chestukhina G.G., Netrusov A.I. (2007). Antimicrobial activity of different proteins and their fragments from *Bacillus thuringiensis* parasporal crystals against clostridia and archaea. Anaerobe.

[86] Silo-Suh L.A., Stabb E.V., Raffel S.J., Handelsman J. (1998). Target range of Zwittermicin A, an aminopolyol antibiotic from *Bacillus cereus*. Curr. Microbiol..

[87] Béchet M., Caradec T., Hussein W., Abderrahmani A., Chollet M., Leclère V., Dubois T., Lereclus D., Pupin M., Jacques P. (2012). Structure, biosynthesis, and properties of kurstakins, nonribosomal lipopeptides from *Bacillus* spp.. Appl. Microbiol. Biotechnol..

[88] El Arbi A., Rochex A., Chataigné G., Béchet M., Lecouturier D., Arnauld S., Gharsallah N., Jacques P. (2016). The Tunisian oasis ecosystem is a source of antagonistic *Bacillus* spp. producing diverse antifungal lipopeptides. Res. Microbiol..

[89] Abderrahmani A. (2011). Identification du Mécanisme de Biosynthèse Non-Ribosomique d’un Nouveau Lipopeptide, la Kurstakine et Etude de son Influence sur le Phénotype de Souches de *Bacillus thuringiensis* Isolées en Algérie. Ph.D. Thesis.

[90] Roh J.Y., Liu Q., Choi J.Y., Wang Y., Shim H., Xu H.G., Choi G.J., Kim J.C., Je Y.H. (2009). Construction of a recombinant *Bacillus velezensis* strain as an integrated control agent against plant diseases and insect pests. J. Microbiol. Biotechnol..

[91] Liu Q., Roh J.Y., Wang Y., Choi J.Y., Tao X.Y., Kim J.S., Je Y.H. (2012). Construction and characterisation of an antifungal recombinant *Bacillus thuringiensis* with an expanded host spectrum. J. Microbiol..

[92] Abdalla M.Y., Al-Rokibah A., Moretti A., Mulè G. (2000). Pathogenicity of toxigenic *Fusarium proliferatum* from date palm in Saudi Arabia. Plant Dis..

[93] Flood J. (2006). A review of fusarium wilt of oil palm caused by *Fusarium oxysporum* f. sp. *elaeidis*. Phytopathology.

[94] Abdullah S.K., Asensio L., Monfort E., Gomez-Vidal S., Salinas J., López Lorca L., Jansson H. (2009). Incidence of the two date palm pathogens, *Thielaviopsis paradoxa* and *T. punctulata* in soil from date palm plantations in Elx, South-East Spain. J. Plant Prot. Res..

[95] Saeed E.E., Sham A., El-Tarabily K., Abu-Elsamen F., Iratni R., AbuQamar S.F. (2016). Chemical control of black scorch disease on date palm caused by the fungal pathogen *Thielaviopsis punctulata* in United Arab Emirates. Plant Dis..

[96] Yang F., Jacobsen S., Jorgensen H.J., Collinge D.B., Svensson B., Finnie C. (2013). *Fusarium graminearum* and its interactions with cereal heads: Studies in the proteomics era. Front. Plant Sci..

[97] Lazreg F., Belabid L., Sanchez J., Gallego E., Garrido-Cardenas J.A., Elhaitoum A. (2013). First report of *Fusarium redolens* as a causal agent of Aleppo pine damping-off in Algeria. Plant Dis..

[98] Lazreg F., Belabid L., Sánchez J., Gallego E. (2016). Root rot and damping-off of Aleppo pine seedlings caused by *Pythium* spp. in Algerian forest nurseries. J. For. Sci..

[99] Mohammed A.S., Kadar N.H., Kihal M., Henni J.E., Sanchez J., Gallego E., Garrido-cardenas J.A., Ahmed O., Bella B., Naouer E.M. (2016). Characterization of *Fusarium oxysporum* isolates from tomato plants in Algeria. Afr. J. Microb. Res..

[100] Zemouli-Benfreha F., Djamel-eddine H. (2014). Fusarium wilt of chickpea (*Cicer arietinum* L.) in North-West Algeria. Afr. J..

[101] Thammasittirong A., Attathom T. (2008). PCR-based method for the detection of *cry* genes in local isolates of *Bacillus thuringiensis* from Thailand. J. Invertebr. Pathol..

[102] He J., Wang J., Yin W., Shao X., Zheng H., Li M., Zhao Y., Sun M., Wang S., Yu Z. (2011). Complete genome sequence of *Bacillus thuringiensis* subsp. *chinensis* strain CT-43. J. Bacteriol..

[103] Zhu Y., Shang H., Zhu Q., Ji F., Wang P., Fu J., Deng Y., Xu C., Ye W., Zheng J. (2011). Complete genome sequence of *Bacillus thuringiensis* serovar *finitimus* strain YBT-020. J. Bacteriol..

[104] Guan P., Ai P., Dai X., Zhang J., Xu L., Zhu J., Li Q., Deng Q., Li S., Wang S. (2012). Complete genome sequence of *Bacillus thuringiensis* serovar *sichuansis* strain MC28. J. Bacteriol..

[105] Murawska E., Fiedoruk K., Bideshi D.K., Swiecicka I. (2013). Complete genome sequence of *Bacillus thuringiensis* subsp. *thuringiensis* strain IS5056, an isolate highly toxic to *Trichoplusia ni*. Genome Announc..

[106] Zhu L., Peng D., Wang Y., Ye W., Zheng J., Zhao C., Han D., Geng C., Ruan L., He J. (2015). Genomic and transcriptomic insights into the efficient entomopathogenicity of *Bacillus thuringiensis*. Sci. Rep..

[107] Yu X., Zheng A., Zhu J., Wang S., Wang L., Deng Q., Li S., Liu H., Li P. (2011). Characterization of vegetative insecticidal protein *vip* genes of *Bacillus thuringiensis* from Sichuan Basin in China. Curr. Microbiol..

[108] Travers R.S., Martin P.A.W., Reichelderfer C.F. (1987). Selective process for efficient isolation of soil *Bacillus* spp.. Appl. Environ. Microbiol..

[109] Paik D.H., Bae S.S., Park H.S., Pan G.J. (1997). Identification and partial characterization of tochicin, a bacteriocin produced by *Bacillus thuringiensis* subsp *tochigiensis*. J. Ind. Microbiol. Biotechnol..

[110] Jiménez-Esquilín A.E., Roane T.M. (2005). Antifungal activities of actinomycete strains associated with high-altitude sagebrush rhizosphere. J. Ind. Microbiol. Biotechnol..

[111] Stephen J., Cavalieri S.J., Ronald J., Harbeck R.J., McCarter Y.S., Ortez J.H., Rankin I.D., Sautter R.L., Sharp S.E., Spiegel C.A., Marie B., Coyle M.B. (2005). Manual of Antimicrobial Susceptibility Testing.

[112] Knaak N., Rohr A.A., Fiuza L.M. (2007). In vitro effect of *Bacillus thuringiensis* strains and Cry proteins in phytopathogenic fungi of paddy rice-field. Braz. J. Microbiol..

[113] Ferrandis M.D., Juárez-Pérez V.M., Frutos R., Bel Y., Ferré J. (1999). Distribution of *cryl*, *cryll* and *cryV* Genes within *Bacillus thuringiensis* isolates from Spain. Syst. Appl. Microbiol..

[114] Crickmore N., Zeigler D.R., Schnepf E., Van Rie J., Lereclus D., Baum J., Bravo A., Dean D.H. *Bacillus thuringiensis* Toxin Nomenclature. http://www.lifesci.sussex.ac.uk/home/Neil_Crickmore/Bt/vip.html.

